# AtEXT3 is not essential for early embryogenesis or plant viability in Arabidopsis

**DOI:** 10.1111/nph.18452

**Published:** 2022-09-21

**Authors:** Nicolas Max Doll, Eduardo Berenguer, Jekaterina Truskina, Gwyneth Ingram

**Affiliations:** ^1^ Laboratoire Reproduction et Développement des Plantes ENS de Lyon, CNRS, INRAE, UCBL F‐69342 Lyon France

**Keywords:** cell wall, cytokinesis, development, embryogenesis, EXTENSIN

EXTENSINs (EXTs) are a subclass of the very diverse family of cell wall proteins known as hydroxyproline‐rich glycoproteins. The EXT subclass is defined by the presence of at least two repeats of the Ser‐Pro_3‐5_ motif in which the proline residues can be hydroxylated and, subsequently, *O*‐glycosylated with arabinose containing oligosaccharides leading to the formation of hydrophilic domains (Cannon *et al*., 2008; Velasquez *et al*., 2011; Ogawa‐Ohnishi *et al*., 2013). In addition, many EXTs from land plants also contain distinct and characteristic hydrophobic Tyr‐containing motifs (Schnabelrauch *et al*., 1996; Held *et al*., 2004; Cannon *et al*., 2008). These have recently been termed cross‐linking (CL)‐EXTs, because of their ability to undergo peroxidase‐mediated covalent cross‐linking *in vitro* through their Tyr residues, a phenomenon that has been proposed to underlie important structural functions in plant cell walls (Marzol *et al*., 2018). EXTENSINs have a marked ability to self‐assemble into dendritic networks *in vitro* (Cannon *et al*., 2008). This capacity, proposed to be based on self‐ordering due to the alternation of hydrophobic and hydrophilic domains (Lamport *et al*., 2011), may mediate observed interactions with other cell wall components, including pectins (Valentin *et al*., 2010).

Despite an abundance of information regarding the biochemical behaviour of EXTs *in vitro*, information on their biological functions *in vivo* remains markedly scarce, possibly due to extensive redundancy between the multiple genes encoding EXTs and EXT‐like proteins within plant genomes. Work in Arabidopsis has identified potential roles for AtEXTs in promoting root‐hair elongation (Velasquez *et al*., 2011), pollen development (Choudhary *et al*., 2015) and early embryogenesis (Hall & Cannon, 2002; Saha *et al*., 2013; Chen *et al*., 2015). This last function is our focus in this Letter.

Following an enhancer‐trap (transposon)‐based mutagenesis screen, Hall & Cannon (2002) isolated a seedling‐lethal mutant called *root‐shoot‐hypocotyl‐defective* (*rsh*) which, when homozygous, produces disorganised embryos that arrest either before (Cannon *et al*., 2008) or just after (Hall & Cannon, 2002) germination. Mutants show clear defects in cell‐plate formation. A transposon insertion in the promoter of the *AtEXT3* gene was stated to be the causal lesion in *rsh* mutants. Consistent with this, complementation of the rsh phenotype using an *AtEXT3* genomic DNA fragment was reported. However, in two subsequent studies, ‘self‐rescue’ of the rsh phenotype to a fully wild‐type phenotype was reported in up to 20% of genetically *atext3* homozygous plants (Saha *et al*., 2013; Chen *et al*., 2015). No intermediate phenotypes were reported, and ‘rescue’ was transmitted stably to all the progeny in subsequent generations. This calls into question the validity of genetic complementation experiments, as the reported ‘self‐rescue’ phenomenon would interfere with this approach. A mechanism involving compensatory regulation of other AtEXT‐encoding genes was evoked to explain this phenomenon. Nonetheless it appears difficult, based on the published data, to exclude the possibility that the reported rsh phenotype is caused by a lesion linked to the original transposon insertion in *AtEXT3*, but affecting a separate locus, and lost by segregation in ‘rescued’ lines.

We became interested in the *AtEXT3* gene as a result of our discovery that the developing embryo of Arabidopsis is covered with an endosperm‐derived ‘sheath’ rich in epitopes detected by anti‐EXT antibodies (Moussu *et al*., 2017). *In silico* data (Le *et al*., 2010) concur with published *in situ* hybridisation data (Francoz *et al*., 2016) in showing that *AtEXT3* is expressed primarily in the embryo‐surrounding endosperm during Arabidopsis seed development, rather than in the embryo itself. We were therefore keen to further investigate the function of AtEXT3 and we generated a series of new alleles in the Col‐0 background using the CRISPR‐Cas9 based technique. We selected four alleles containing deletions or insertions within the 5′ coding sequence of the *AtEXT3* gene (Fig. 1a). All are predicted to produce either strongly truncated or frame‐shifted proteins (Fig. 1b). Being aware of recent work suggesting that 5′ mutations generated by CRISPR can lead to the production of functional proteins due to the use of downstream in‐frame ATGs (Smits *et al*., 2019), we verified the *AtEXT3* coding sequence (which lacks introns) for such sequences. None are present. We therefore conclude that all four alleles are null. Homozygous plants were all fully phenotypically wild‐type in terms of root growth, plant growth and reproductive development (Fig. 1c–e). No defective seeds or seedlings were detected for any line, in any generation.

**Fig. 1:**
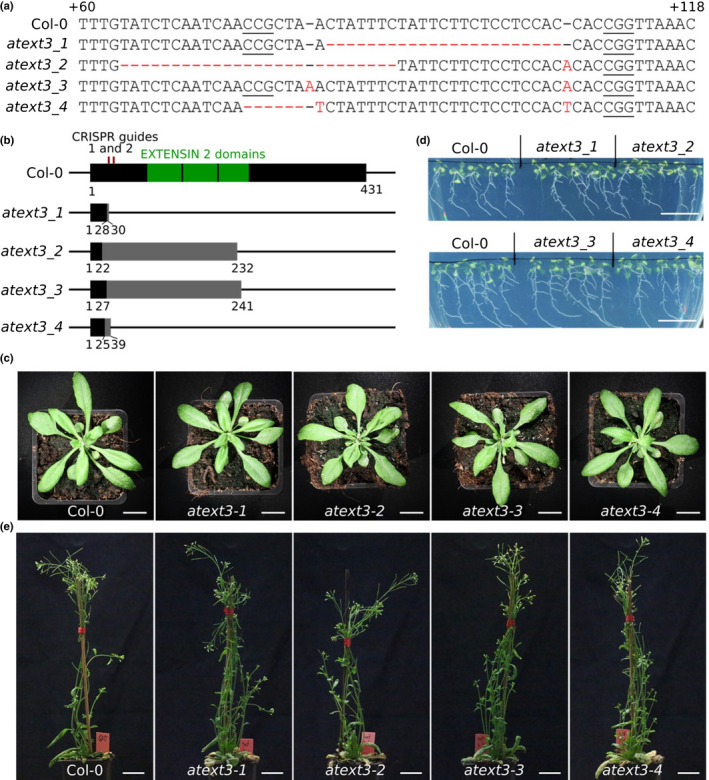
Loss of At*EXT3* function gives no obvious phenotypes in the Arabidopsis Col‐0 background. (a) DNA sequence of the At*EXTENSIN3* (*AT1G21310*) gene between position +60 and +118 from the predicted start of translation. The CRISPR/Cas9 alleles generated are represented below and named at*ext3‐1 to ‐4*. The protospacer adjacent motif (PAM) sequences of the two guides used are underlined. The mutations in the mutant alleles sequence are indicated in red. (b) Predicted consequences of the mutations on the AtEXT3 protein. The 3 EXTENSIN‐2 domains predicted by the Protein Families database (Pfam) are represented as well as the position of the two guides used. For the predicted mutant proteins, sequences that differ from those of Col‐0 due to a frameshift are represented in grey. The amino acid positions from the predicted first methionine are indicated. (c) At 14 d after stratification (DAS) seedlings, grown *in vitro* for Col‐0 and for the four At*EXT3* null alleles. (d) Here, 29 DAS plants grown on soil for Col‐0 and for the four At*EXT3* null alleles. (e) Finally, 46 DAS plants grown on soil for Col‐0 and for the four At*EXT3* null alleles. Bars: (c, d) 2 cm; (e) 3 cm.

Our results suggest that loss of AtEXT3 does not lead to detectable defects in development in the Col‐0 background. However, the *rsh* mutant originally described by Hall and Cannon was identified in the Landsberg *erecta* (L‐*er*) background. To eliminate the possibility that a suppressor exists in Col‐0 that is not present in the L‐*er* background, we carried out CRISPR experiments in the L‐*er* background, this time using guides predicted to interrupt the EXTENSIN 2 domains of the AtEXT3 protein. Of multiple null alleles brought to homozygosity we selected three deletions predicted to produce either strongly truncated or frame‐shifted proteins (Fig. 2). We were again unable to detect any defects in root growth, plant growth or reproductive development in any of these lines. In addition, we confirmed the absence of phenotypes during seed development, and germination (Supporting Information Fig. S1).

**Fig. 2:**
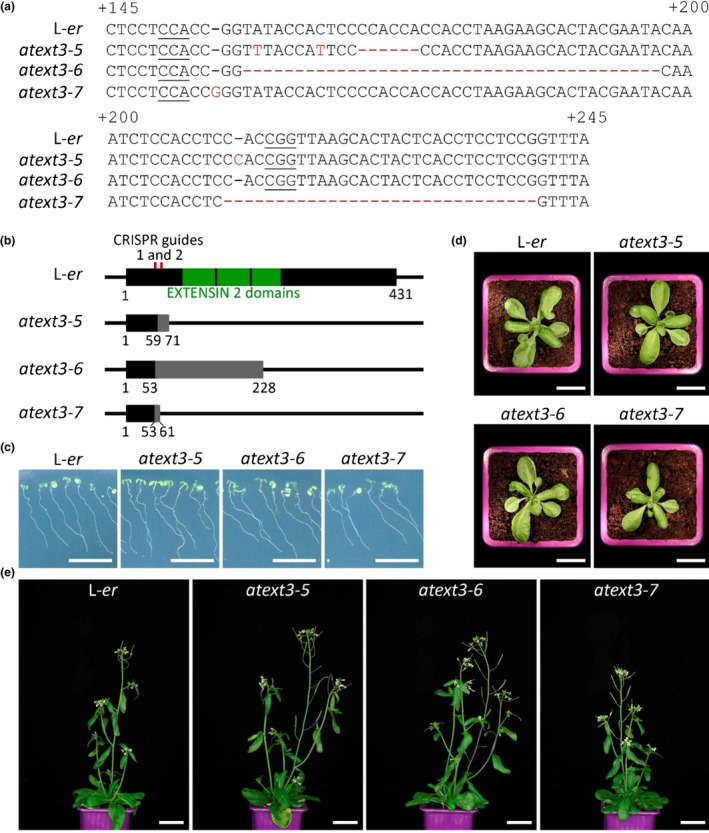
Loss of At*EXT3* function gives no obvious phenotypes in the Arabidopsis L‐*er* background. (a) DNA sequence of the At*EXTENSIN3* (*AT1G21310*) gene between positions +145 and +245 from the predicted start of translation. The CRISPR/Cas9 alleles generated are represented below and named at*ext3‐5 to ‐7*. The protospacer adjacent motif (PAM) sequences of the two guides used are underlined. The mutations in the mutant alleles are indicated in red. (b) Predicted consequence of the mutations on the AtEXT3 protein. The three EXTENSIN‐2 domains predicted by the Protein Families database (Pfam) are represented as well as the position of the two guides used. For the mutant proteins, sequences that differ from L‐*er* due to a shift in the open reading frame (ORF) are represented in grey. The amino acid positions from the predicted first methionine are indicated. (c) At 10 d after stratification (DAS) seedlings, grown *in vitro* for L‐*er* and for the three At*EXT3* null alleles. (d) Here, 28 DAS plants grown on soil for L‐*er* and for the three At*EXT3* null alleles. (e) Finally, 46 DAS plants grown on soil for L‐*er* and for the three At*EXT3* null alleles. Bars: (c, d) 2 cm; (e) 3 cm.

Taken together, our results indicate that AtEXT3 is not essential for embryogenesis and plant development in Arabidopsis, suggesting that its function is likely to be redundant with other related proteins. We feel that it is important that our result be communicated to the scientific community, as the role of AtEXT3 has been very widely cited as a proof of the important function of EXTs in plant development and particularly in cytokinesis. Although fully convinced that EXTs are of primary importance in plants, we propose that the roles of AtEXT3 may be almost fully redundant with those of other related proteins. Supporting this hypothesis, phenotypeless *atext3* mutants were shown to have extensive changes in the expression of other AtEXT‐encoding genes, or the accumulation of AtEXT proteins (Saha *et al*., 2013; Chen *et al*., 2015). It is possible that a similar phenomenon occurs in the CRISPR generated *atext3* mutants.

Finally, our study does not call into question the presence of AtEXT3 in cell walls post germination, or the beautiful biochemical studies of AtEXT3 behaviour *in vitro* that have been published (Cannon *et al*., 2008).

## Materials and Methods

### CRISPR/Cas9 allele generation

The CRISPR/Cas9 alleles of At*EXT3* (*AT1G21310*) were generated through the method described by Wang *et al*. (2015) for double guides assembly. The two guides were designed on the Crispr‐P v2.0 software and chosen to target the 5′ end of the gene (first guide, 5′‐GAAGAATAGAAATAGTTAG‐3′; second guide, 5′‐TATTCTTCTCCTCCACCAC‐3′). For the cloning, the primers used were 5′‐ATATATGGTCTCGATTGGAAGAATAGAAATAGTTAGGTT‐3′ and 5′‐TGGAAGAATAGAAATAGTTAGGTTTTAGAGCTAGAAATAGC‐3′ for the first guide and 5′‐AACGTGGTGGAGGAGAAGAATACAATCTCTTAGTCGACTCTAC‐3′ and 5′‐ATTATTGGTCTCGAAACGTGGTGGAGGAGAAGAATACAA‐3′ for the second guide. The pHEE401 plasmid was used as the destination vector.

For the generation of the CRISPR/Cas9 alleles of *EXT3* on the L‐*er* background the method described by Stuttmann *et al*. (2021) was used. Two guides (first guide, 5′‐GGTGGGGAGTGGTATACCGG‐3′; second guide, 5′‐ATACAAATCTCCACCTCCAC‐3′) were inserted into the pDGE332 and pDGE334 shuttle vectors and recombined into the pDGE347 recipient vector.

### Stable transformation of *Arabidopsis thaliana* (L.) Heynh

The plasmids generated were first transformed into the C58PMP90 *Agrobacterium* strain by electroporation at 2.2 kV in a 1‐ml cuvette (Eurogentec, Liege, Belgium). Agrobacteria were then grown at 28°C for 2 h in LB liquid medium without antibiotics before being spread on Petri dishes containing YEB solid medium with rifampicin (50 mg l^−1^), gentamicin (20 mg l^−1^) and kanamycin (50 mg l^−1^) for the pHEE401‐based plasmid or rifampicin (50 mg l^−1^), gentamicin (20 mg l^−1^) and spectinomycin (100 mg l^−1^) for the pDGE347 based plasmid. After 2 d, the agrobacteria were used to transform Col‐0 plants using the floral‐dip method (Logemann *et al*., 2006). Selection of transgenic plants was performed on Murashige & Skoog (MS) plates with 30 mg l^−1^ of hygromycin.

### Identification of the mutant alleles and generation of homozygous plants

Amplification of the region around the cutting sites was performed by PCR on DNA extracted from transgenic Col‐0 plants using the 5′‐GGGTGTGAAGGGAAGGCACTAAATC‐3′ and 5′‐GTAAACGTAGTGCTTCTTTGGTGG‐3′ primers. For the transgenic L‐*er* plants the primers used were 5′‐GGGTGTGAAGGGAAGGCACTAAATC‐3′ and 5′‐AGGTGGGGGTGGGGAATGGTA‐3′. Sequencing was carried out with the 5′‐GTAAACGTAGTGCTTCTTTGGTGG‐3′ (Col‐0) or and 5′‐GGGTGTGAAGGGAAGGCACTAAATC‐3′ (L‐*er*) primers. Homozygous mutants for *atext3* were selected from the T2 plants. In the Col‐0 background the CRISPR/Cas9 cassette was removed by selection of hygromycin sensitive plants and validated by the absence of cassette amplification by PCR with the 5′‐TGTCCCAGGATTAGAATGATTAGGC‐3′ and 5′‐AGCCCTCTTCTTTCGATCCATCAAC‐3′ primers. Homozygous mutants were confirmed by sequencing. In L‐*er* transgenic plants nonfluorescent seeds were selected in the T2 and homozygous mutants were confirmed by sequencing.

### Plant growth

For the selection processes and *in vitro* growth (Figs 1c, 2c), seeds were gas sterilised with chlorine gas (3 ml of HCl (33%) in 100 ml of bleach) for at least 3 h in a hermetic box. Seeds were then sown on MS medium with 0.5% of sucrose and, for the selection of Col‐0 mutants, with 30 mg l^−1^ of hygromycin. Stratification was carried out 2 d at 4°C in darkness. Plates were then transferred into growth chambers in long day conditions (16 h light, 21°C). For the *in vitro* growth experiments, plants were grown for 14 d (Col 0) or 10 d (L‐*er*) and pictures were taken with an Epson perfection V300 photo scanner. For germination tests, plants were grown for 12 d (100 seeds per plate). In all the other cases, seedlings were transferred after 7 d onto soil (Argile 10 (favorit)) and grown in long day conditions (16 h light, 21°C). Images of plants at 29 d after stratification (DAS) and 46 DAS (Fig. 1d,e) or 28 DAS and 46 DAS (Fig. 2d,e) were taken with a Canon EOS 450D camera with a Sigma 50 mm f/2.8 DG Macro objective. For analysis of silique filling, individual siliques were opened at the mature green stage, and seeds were counted under a binocular microscope.

### Seed clearing

To visualise developing seeds, the siliques at 7 d after pollination (DAP) were dissected with a needle and forceps on adhesive tape on a microscope slide and mounted in the clearing solution (1 : 7, glycerol : chloral hydrate liquid solution, v/v; VWR Chemicals, Rosny‐sous‐Bois, France). After 48 h incubation at 4°C samples were imaged under a Zeiss Axio Imager M2 microscope.

## Competing interests

None declared.

## Author contributions

GI and NMD led the study. GI obtained funding for the study. GI supervised the work. NMD, EB and JT carried out the experiments. GI and NMD wrote the paper with input from all authors. NMD and EB contributed equally to this work.

## Supporting information


**Fig. S1** *atext3* mutants show no obvious phenotype during seed development or germination.
Please note: Wiley Blackwell are not responsible for the content or functionality of any Supporting Information supplied by the authors. Any queries (other than missing material) should be directed to the *New Phytologist* Central Office.

## Data Availability

The data that support the findings of this study are available from the corresponding author upon reasonable request.
