# Supplementary material for: AtEXT3 is not essential for early embryogenesis or plant viability in Arabidopsis
Source: New Phytol. 2022 Sep 21;236(5):1629–33. doi: 10.1111/nph.18452 (PMC9826179; doi:10.1111/nph.18452)
Supplement: Supplementary file 1 — Fig. S1 atext3 mutants show no obvious phenotype during seed development or germination. Please note: Wiley Blackwell are not responsible for the content or functionality of any Supporting Information supplied by the authors. Any queries (other than missing material) should be directed to the New Phytologist Central Office. [file NPH-236-1629-s001.zip › NPH_18452_Supporting Information Fig. S1.pdf]

New Phytologist Supporting Information Article Title: AtEXT3 is not essential for early embryogenesis or plant viability in Arabidopsis.

Authors: Nicolas Max Doll, Eduardo Berenguer, Jekaterina Truskina and Gwyneth Ingram

Article acceptance date: 25/08/2022

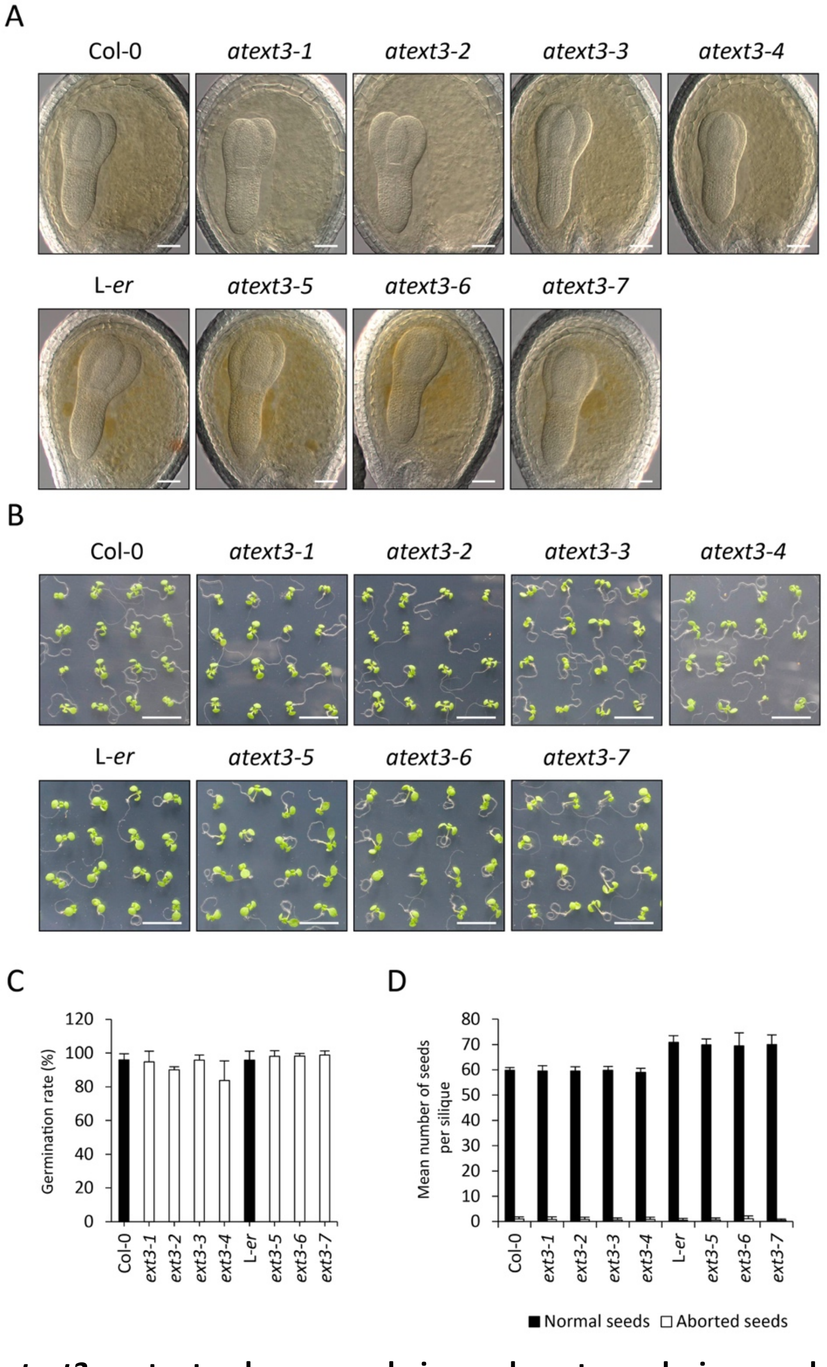

Figure S1: *atext3* mutants show no obvious phenotype during seed development or germination.

A) Clearing of representative developing seeds from indicated genotypes 7 days after pollination (DAP). No evidence of early seed abortion was observed in any genotype. Scale bars 50 µm. B) 12-day-old seedlings, grown *in vitro* in MS medium with 0.5% sucrose. Scale bars 1 cm. C) Quantification of germination rates from samples shown in B). Histogram of mean germination rates obtained from 4 biological replicates, each containing 100 seeds. Error bars show standard deviations. ANOVA revealed no difference between means. D)

Analysis of seed number per silique in indicated genotypes. No evidence of early seed abortion was observed. 6 siliques from 2 individuals were quantified for each genotype. Error bars indicate standard deviations.
